# Impaired Social Attention and Cognitive Empathy in a Paediatric Sample of Children with Symptoms of Anxiety

**DOI:** 10.1007/s10802-024-01240-7

**Published:** 2024-09-18

**Authors:** Steve Eaton, Ellie Mae Dorrans, Stephanie H.M. van Goozen

**Affiliations:** 1 Neurodevelopment Assessment Unit (NDAU), Centre for Human Developmental Science (CUCHDS), School of Psychology, Cardiff University, 70 Park Place, Cardiff, CF10 3AT UK; 2 Department of Clinical Neurodevelopmental Studies, Leiden University, Leiden, the Netherlands

**Keywords:** Anxiety, Empathy, Eye-tracking, Children, RDoC

## Abstract

Impairments in social cognition, in particular empathy, have been associated with childhood psychopathology, though previous investigations have yielded inconsistent results. Measures of social attention can reveal processes involved in responses to emotional stimuli and highlight deficits in empathy, or emotional biases in those with anxiety. The current study examined symptoms of anxiety, cognitive and affective empathy scores, and eye-gaze patterns in a pediatric sample of children (*n* = 178; 51–98 months-old) referred by their teachers for emerging psychopathology symptoms at school. We used eye-tracking metrics to capture gaze patterns during a dynamic video task designed to elicit empathic responses. Anxiety symptomology was reported by parents using the Screen for Child Anxiety Related Disorders scale (SCARED). Associations between eye-tracking variables, cognitive and affective empathy, and anxiety scores were analysed dimensionally in accordance with the Research and Domain Criteria (RDoC) framework. Higher levels of anxiety were associated with lower cognitive empathy and shorter first and total fixation durations to the eyes, across emotions (happiness, sadness, fear). No such associations were found between affective empathy and anxiety. Hierarchical multiple regression analyses revealed that across emotion conditions, first fixation duration negatively predicted anxiety scores. Our results indicate that children high in anxiety display cognitive empathy impairments and shorter attention to the eyes. These findings could inform early intervention programs for individuals at risk of developing anxiety disorders, as educating those high in anxiety on ways to identify emotions in others through changes in social attention could help to reduce anxiety.

**Supplementary Information:**

The online version contains supplementary material available at 10.1007/s10802-024-01240-7.

## Introduction

Anxiety disorders are among the most common psychiatric disorders affecting children. Relative to other disorders they typically emerge early in development (Kessler et al., 2005), and are a risk factor for the development of other mental illnesses in adulthood (Cartwright-Hatton et al., 2006). Anxiety is a broad term encompassing several subtypes including separation anxiety (excessive fear felt by sufferers regarding the loss of an attachment figure), social anxiety (fear of embarrassment in social situations), and generalized anxiety disorder (GAD; regular and uncontrollable worries over a wide range of things in life). However, a common factor linking these subtypes is a heightened sensitivity to perceived threat (Dudeney et al., 2015). This can manifest as an attentional bias toward potentially threatening information, resulting in an increased likelihood that an anxious individual will interpret a neutral situation as threatening (Puliafico & Kendall, 2006). Accordingly, threat-related attentional biases in anxious individuals have been consistently demonstrated in the literature across a range of experimental paradigms (Bar-Haim et al., 2007), and such biases are a key mechanism for both the maintenance and aetiology of anxiety disorders (Lichtenstein-Vidne et al., 2017).

However, both the Bar-Haim et al. and Lichtenstein-Vidne et al. studies used disorder categories – informed by DSM criteria – to identify those with anxiety. More recently, transdiagnostic approaches to understanding mental health difficulties, characterised by dimensional measures of domains of functioning, have been proposed as a more reliable alternative to the categorical classification of psychiatric disorders (Dalgleish et al., 2020). The Research Domain Criteria (RDoC) approach, established by the National Institute of Mental Health, identifies five functional domains (negative valence systems, positive valence systems, cognitive systems, social processes, and arousal / regulatory systems) that can be used to examine psychological constructs relevant to psychopathology dimensionally, rather than focusing on discrete disorder categories only (Insel et al., 2010). The RdoC framework places anxiety under the ‘negative valence systems’ domain, where it is conceptualised as ‘potential threat’. Applying the RdoC framework to investigations of childhood anxiety disorders can advance understanding of their potential causes and shed light on potential treatments (Lebowitz et al., 2018).

Social cognition is a term describing a cluster of mental processes that an individual uses to understand – and apply information about – social interactions. A component of social cognition, empathy, refers to the accurate comprehension of another person’s emotional state (Decety & Jackson, 2004). Successfully recognizing and understanding emotional states in others is an aspect of emotion understanding, which can be defined as an individual’s ability to understand the nature, cause, and regulation of emotion, either in themselves or others, and is therefore closely linked to social cognition and empathy (Pons et al., 2004; Sprung et al., 2015).

Empathy allows an individual to adjust their behavioural responses in ways that are appropriate to the situation and another person’s needs. It can therefore promote cooperation and group adhesion and accordingly, has been found to play an important role in prosocial behaviours (Jolliffe & Farrington, 2006). From a research perspective, empathy can be subdivided into two dissociable processes – cognitive empathy (recognising and understanding the emotions experienced by others), and affective empathy (vicariously experiencing the emotions of another; Blair, 2005). Cognitive empathy can be considered an aspect of emotion understanding, as it requires an individual to recognise and understand the possible causes of others’ expressed emotions.

Impairments in appropriate empathic responses have been identified in a range of psychiatric disorders in children (Decety & Moriguchi, 2007), including externalising disorders such as conduct disorder and attention deficit / hyperactivity disorder (ADHD), and internalising disorders such as anxiety (Pearcey et al., 2021). Given that there are overlapping symptoms between disorders such as ADHD and autism (Mayes et al., 2012), and anxiety and depression (Eysenck & Fajkowska, 2018), an RDoC approach would enable an understanding of potential associations between empathy and psychological constructs that relates to various forms of psychopathology, irrespective of discrete mental disorder categories.

With regards to empathic responses in those with internalising disorders, Gambin and Sharp (2018) identified a positive association between affective empathy and anxiety in adolescents, reasoning that displays of anxiety in others could trigger or enhance anxious states in the observer. The study also found cognitive empathy to be negatively associated with social anxiety and proposed that the ability to recognise and understand another person’s emotions enables contextually appropriate behaviours that help to reduce social anxiety. A meta-analysis of studies examining social anxiety and empathy by Pittelkow et al. (2021) found weak, positive associations between social anxiety and affective empathy, and weak, negative associations between social anxiety and cognitive empathy. However, there was considerable heterogeneity in the study designs and operationalising of key variables, and the authors acknowledged that there are a small number of studies in this area. Such factors threaten the reliability of the findings, and the current body of evidence renders the role of cognitive empathy in the development of anxiety disorders unclear. That is, low cognitive empathy could contribute to the development of anxiety symptoms, or it could be a consequence of anxiety, or it could be a variable that correlates with anxiety but does not influence the intensity of symptoms either way. In contrast, Howe-Davies et al. (2022) found affective empathy to be negatively correlated with anxiety (indexed by a composite score consisting of social anxiety, separation anxiety, and generalized anxiety), and argued that children higher in anxiety can experience social cues as threatening, which impairs their ability to effectively share other people’s emotions.

Taken together, these studies illustrate that although associations between empathy and anxiety likely exist, the precise nature of their relationship is not well-defined, and that there is an emphasis on studies of empathy and *social* anxiety in the literature and correspondingly, a lack of research that examines the subcategories of empathy (i.e., cognitive and affective) in relation to a general measure of anxiety. This is especially relevant when studying young samples, given that some types of anxiety are less prevalent in children (Beesdo et al., 2009).

The propensity for a child to behave in aggressive or antisocial ways as seen in some forms of externalising disorders, has also been proposed as causally linked to empathy impairments through reduced cognitive (impairments in understanding and recognise emotions in others; Frick & Kemp, 2021) and affective (not sharing in the distress of others) empathy (Blair, 2005; Van Goozen et al., 2022). Accordingly, Hunnikin et al. (2020) found that children high in disruptive behaviour were impaired in cognitive and affective empathy, and that those same children also showed impairments in recognising negative emotions. Empathy impairment has also been associated with another form of externalising disorder, ADHD, as those who present as high in symptoms of the inattentive dimension of ADHD (e.g., trouble focusing) struggle to consider events from another person’s perspective (Cordier et al., 2010; Marton et al., 2009).

Investigations of associations between empathy and psychopathology therefore provide mixed results and suggest that there is a lack of a coherent theoretical framework that links findings together, especially across the broad categories of behavioural problems (i.e., externalising and internalising disorders). However, these data more closely cohere when considered through the lens of social attention. In order to recognise and appropriately respond to another person’s emotions, it is first necessary to attend to socially relevant cues. Therefore, social attention is a process that precedes empathic responses, and it is these initial observations that provide information that is critical for accurately decoding and comprehending emotions in others.

If attention to the eyes during face processing is considered a necessary metric for correctly recognizing emotions in faces (Batty et al., 2011; Taylor et al., 2001), and accurate decoding of emotions is necessary for an empathic response, it follows that aberrant eye-gaze patterns would be seen in those low in empathy. Accordingly, associations between gaze patterns and empathy have been examined, with Cowan et al. (2014) reporting that greater affective empathy in adults was associated with longer fixation on the eyes of a person relaying a fictional personal event, and Moutinho et al. (2021) finding that eye region fixations positively correlated with a self-reported measure of empathy in healthy adults. Few studies examine empathy and eye-tracking metrics in children; however, van Zonneveld et al. (2017) compared children (8–12 years) at high risk of developing criminal behaviour to a typically developing control group and found that the at-risk group showed impaired affective empathy, intact cognitive empathy and no differences with the control group in terms of eye-gaze patterns.

As well as empathy, eye-tracking metrics can also reveal associations between internalising and externalising symptoms and gaze patterns. In a study of children aged 9–13 years, Michalska et al. (2017) found anxiety symptoms to be correlated with eye gaze avoidance, a finding that supports those reported in similar studies of anxious adults (e.g., Moukheiber et al., 2010). Similarly, when participants were presented with three images depicting non-social objects and one image depicting the eye region of a face, Kleberg et al. (2017) found anxiety in adolescents was associated with a reluctance to orient toward the image of the eyes.

A study of gaze patterns by Dadds et al. (2011) revealed that males high in callous-unemotional traits showed diminished eye-contact with their parents, and this reduction was associated with low trait empathy. Similarly, using an emotional face processing task, Menks et al. (2021) found that those higher in conduct disorder (CD) traits spent less time attending to the eyes than a typically developing comparison group, irrespective of the facial stimuli presented (i.e., negative / neutral expression), and an examination of ADHD and eye gaze by Airdrie et al. (2018) found that compared to healthy controls, those higher in ADHD symptoms spent less time fixating on the eyes in an emotion recognition task. However, Hunnikin et al. (2020) found no differences in social attention to the eyes between typically developing children and children with disruptive behaviour disorders.

Therefore, eye gaze patterns have been found to be similar in investigations of behavioural disorders, suggesting that those high in externalising *and* internalising symptoms avoid attending to the eyes. Nevertheless, disparate approaches to operationalising gaze patterns were used across these studies, with the Hunnikin et al. and Airdrie et al. (2018) studies both using percentage of dwell time to the eyes (calculated by summing all fixations to the eyes and dividing by the total duration of time spent looking at the face, the Menks et al. (2021) study using number of fixations to the eyes, the Michalska et al. study using fixation duration and fixation count, the Kleberg et al. (2017) study using an image of the eye region as a stimulus that was presented alongside other stimuli not associated with facial processing, and the Dadds et al. (2011) study coding for eye-contact between family members during a ‘free play’ task paradigm. Additionally, the samples in these studies were mostly adolescents and some contained a disproportionate number of male or female participants.

The literature examining adults suggests that gaze patterns characterised by avoiding the eyes of others – reflecting patterns seen in studies of individuals high in psychopathology symptoms – are associated with impaired empathic responses. However, there is a lack of studies examining gaze patterns and empathy in children, and the extant literature investigating empathy and social attention contains several gaps, as it does not examine cognitive and affective empathy subtypes separately.

The aim of the present study was therefore to examine social attention, and cognitive and affective empathy, in a sample of young children with symptoms of anxiety using an RDoC perspective. That is, the construct of ‘Potential threat’ (anxiety), under the Negative Valence Systems domain, will be examined using the physiology (eye-tracking), and self-report (empathy quotient) units of analysis (Insel et al., 2010), and all measures included in the study are dimensional and not bound by diagnostic rubric. We first aimed to examine relationships between cognitive and affective empathy and childhood anxiety in a sample of young children with emerging emotional and behavioural problems. We hypothesised that children with higher levels of anxiety would show greater impairments in empathy. Due to contrasting findings in previous studies, we explored associations between these variables rather than proposing specific and directional hypothesis that predicted them. We then sought to characterize any identified associations by examining social attention using eye-tracking metrics. Given previous literature in this area, and literature describing threat-related biases in those high in anxiety (e.g., Bar-Haim et al., 2007), our hypothesis regarding eye gaze patterns was that children higher in anxiety symptoms and lower in empathy would show an aversion to the eyes when viewing negative stimuli.

In addition to these aims, the present study sought to build upon previous research from our lab. Howe-Davies et al. (2022) examined cognitive and affective empathy, and cognitive and affective theory of mind (ToM), in a similar sample. However, measures of social attention were not included in the study, meaning that it is unclear if attentional processes could help to explain the findings. As such, the present study looked to further explore relationships between cognitive and affective empathy using eye-tracking data. Because a greater number of girls than boys are likely to suffer from internalizing disorders (Mayes et al., 2020), and because empathy is a social skill that develops over time (Decety & Michalska, 2010), we also explored the roles of gender and age in any associations found between anxiety and empathy.

## Method

### Participants

The sample consisted of one hundred and seventy-eight children (63 females) aged 51–98 months (M_age_=77.17) who were referred to the Neurodevelopment Assessment Unit (NDAU; https://www.cardiff.ac.uk/neurodevelopment-assessment-unit) at Cardiff University by their schools for an assessment due to the presentation of emotional, cognitive, or behavioral difficulties. The sample therefore contains a heterogeneous range of emotional and / or behavioral problems. None of the children had received a diagnosis at the time of testing, though some were on a diagnostic pathway. Written informed consent was obtained from the parent or caregiver for each child prior to the assessment and all experimental procedures were approved by the relevant university ethics committee (EC.16.10.11.4592GR). Due to the difficulties associated with assessing young children with emerging behavioural problems, we were unable to obtain eye-tracking data from the entire sample. Therefore, the number of participants examined in each individual analysis varied. However, differences in the number of participants in each assessment was small, ranging only from *N* = 159 – *N* = 178.

### Testing Procedure

Participants and parents were assessed by trained graduate researchers, and tasks were administered in a fixed order for each child. At the same time, in a separate room, parents completed a diagnostic interview and a battery of self-completion questionnaires regarding their child’s behaviors over the previous 6 months.

### Anxiety Measure

The Screen for Child Anxiety Related Emotional Disorders (SCARED; Birmaher et al., 1997) is a parent- and youth-reported questionnaire developed to screen youth for anxiety disorders as outlined by the Diagnostic Statistical Manual, Fourth Edition (DSM-IV; American Psychiatric Association, 1994). Responses are rated on a scale of 0–2 and yield 5 subscale scores (generalized anxiety disorder, social phobia, separation anxiety disorder, panic / somatic disorder, and school phobia), and a total score. A total score of ≥ 25 may indicate the presence of an anxiety disorder. The SCARED has been found to be reliable in terms of internal consistency (Muris & Steerneman, 2001), and discriminant validity (Birmaher et al., 1999), and strong in terms of test-retest reliability (Behrens et al., 2019). Although the SCARED is often used in children aged 8-years and above, its’ consistency and validity as a measure of anxiety in younger children (aged 4-7-years) has recently been demonstrated (Scoberg et al., 2024). The SCARED questionnaire was completed by the parents of the participants as part of their questionnaire battery. The internal consistency of the SCARED total score was assessed by calculating a Cronbach α coefficient. The coefficient value was 0.94, suggesting high reliability and strong internal consistency.

### Empathy Measurement

Participants viewed three 50s film clips to induce an empathic response. These were first used in a study by Noten et al. (2019) which showed that the emotions presented in the clips could be recognized and understood by children as young as three-years-old. The clips were selected due to their authenticity (children displaying real-life (i.e., not acted) emotions, and because using dynamic – as opposed to static – stimuli is important when studying social attention (Noten et al., 2019). One clip represented happiness (a boy opens a present at Christmas), a second represented sadness (a boy flushes his dead goldfish down the toilet), and a third represented fear (a girl is afraid of being in a car wash). Following each clip, the children were first questioned about the emotions they believe were displayed by the protagonist in the clip (assessment of cognitive empathy) through an open question (i.e., “How was that little boy feeling?”), which was followed by a further question that explored why the child believed the protagonist felt that way. A further open question was asked (i.e., “Was he feeling anything else?”), which was again followed by a question that allowed the child to explain their response. If the child was unsure, they were offered a list of potential emotions that could prompt a response (i.e., “Were they feeling angry / sad / happy / scared / excited?”). In the same way, the children were then asked about the emotions they themselves experienced whilst viewing the clip (assessment of affective empathy). To score the children’s cognitive and affective responses, a coding system adapted from Strayer (1993) was used. For details on cognitive empathy see Braaten and Rosén (2000), and for details on affective empathy, see Strayer (1993). For cognitive empathy, ‘target’ emotions (i.e., the correct emotion) and ‘relevant’ emotions were coded as 0 for an incorrect response and 1 for a correct response. Interpretations of these emotions were then coded as 0 = no match (irrelevant or no response), 1 = egocentric interpretation (the participant referred to their own emotional state but not the episode itself), 2 = situation-centred interpretation (the participant referred to the situation but not to the feelings of the character), and 3 = character-centred interpretation (the participant provided an explanation that referenced the character’s feelings or experience). Cognitive empathy scores therefore ranged from 0 to 8. For affective empathy, emotions experienced by the participants were also divided into ‘target’ and ‘relevant’ emotions, and were coded as 0 = no match, 1 = similar valence (the participant experienced an emotion similar in valence to that of the character in the video), 2 = same emotion / different intensity (the participant experienced an emotion the same as the character’s, but at a different intensity), and 3 = same emotion / same intensity (the participant’s emotion and intensity were the same as the character in the video). Affective empathy scores therefore ranged from 0 to 6, with higher scores reflecting better empathy (see also Howe-Davies et al., 2022).

Inter-rater reliability was established using an intraclass correlation coefficient (ICC). The ICC ranges from 0 to 1 with values less than 0.50 considered poor, between 0.50 and 0.75 moderate, between 0.75 and 0.90 good, and above 0.90 considered excellent (Koo & Li, 2016). Three coders independently viewed and scored each video clip, and any discrepancies were resolved through discussion between the coders. The final intraclass correlation coefficient showed good to excellent reliability between raters (affective empathy = 0.89; cognitive empathy = 0.93).

### Externalising Symptoms Measure

The Child Behaviour Checklist (CBCL; Achenbach et al., 2003) was used to assess externalising symptoms. Due to the age range of the sample, both the preschool (1.5–5-years) and child (6–18-years) were used. Parents rated their children on a three-point scale consisting of 118 items. Behaviours were rated as ‘0’ for not present, ‘1’ if the child sometimes demonstrated the symptom, and ‘2’ if the symptom was consistently demonstrated. Raw scores were converted to standardised (T) scores based on the child’s age and sex, which were then used as an index of symptom severity (scores > 69 are considered clinically relevant (Achenbach & Rescorla, 2001). The CBCL is widely-used and has been shown to be a reliable and well-validated measure of childhood psychiatric symptoms (Nakamura et al., 2009). Cronbach’s α coefficient for CBCL externalising symptoms was 0.87 suggesting good reliability and internal consistency.

### Eye-Tracking Apparatus

Eye movements were recorded using a Tobii X2-60 binocular eye-tracker at 60 Hz sampling frequency. The film clips were presented on a monitor with a 22-inch screen with a resolution of 1680 × 1080 pixels. The eye-tracking device was adhered beneath the monitor of the laptop, approximately 60 cm from the participant’s eyes in a dimly lit room. Prior to the presentation of each clip, calibration was performed so that the tracker could precisely monitor the participant’s eye movements. Calibration was a five-point process consisting of the participant being asked to fixate on the upper-left corner of the screen, followed by the lower-left, upper-right, lower-right, and centre. Results were displayed to the researcher immediately following calibration, so that they could either accept the calibration accuracy, or request that the participant repeat the calibration process to improve accuracy. Successful fixation on all areas was required for the assessment to begin.

Social attention was measured using four eye-tracking metrics: (1) Time to First Fixation (TTFF), describes the amount of time before the participants fixates on the area-of-interest and represents a top-down, attention driven search; (2) First Fixation Duration (FFD), describes how long the participants fixates on the area-of-interest once they initially fixate upon it, and therefore provides data on how long an area of interest holds a participant’s attention. When used alongside TTFF, FFD can reveal how much an area of interest attracted attention (e.g., a short TTFF and long FFD suggests a great deal of interest in an area); (3) Total Fixation Duration (TFD), describes how long the participant focused on the area-of-interest for the duration of the clip, and provides an understanding of the amount of cognitive effort given to paying attention to an area by the participant; and (4) Fixation Count, which describes the number of times the participants focused on the area of interest throughout the presentation of the clip, and can be used to determine the importance a participant places on an area of interest.

For all clips, the area-of-interest was the eyes, as this region has been identified as key in social cognition (Itier & Batty, 2009). As the size of the protagonist was different in each clip (i.e., they were closer to or further away from the viewer), the size of the area-of-interest varied for each clip. The area-of-interest was an oval within a rectangle that was sized 230 × 92 pixels (happy film clip), 227 × 85 pixels (sad film clip), and 368 × 152 pixels (fear film clip).

### Statistical Analyses and Data Screening

All statistical analyses were carried out using the Statistical Package for Social Sciences (IBM SPSS 24) software. Unless otherwise stated, the alpha level for significance was set at 0.05. Normality of the data was tested using the Kolmogorov-Smirnov test for normality (Mishra et al., 2019). With the exception of age, all tests were significant (*p* < .001), meaning the data were non-normally distributed. Despite this, outliers in the SCARED data were not removed in order to normalise the data for analyses, as we were interested in the attentional processes in those higher and lower in anxiety and so needed to include those at both ends of the distribution.

Associations between empathy and anxiety, and between eye-tracking metrics and empathy and anxiety, were tested for. To examine the contributory effects of cognitive and affective empathy, and social attention, on anxiety symptoms, three hierarchical multiple regression analyses were conducted (one for each emotion), whilst controlling for the possible effects of gender, age, and externalising symptoms.

## Results

### Preliminary Analyses

Table 1 displays descriptive information for age, CBCL internalizing and externalizing subscale scores, SCARED scores, and cognitive and affective empathy scores for the whole sample, and also by gender. Table 1 also provides test statistics for comparisons between the males and females in terms of age, SCARED scores, and psychopathology symptoms. Independent samples *t*-tests revealed no significant differences between males and females in terms of age, SCARED total scores, CBCL internalising and externalising scores. Differences between males and females with regards to empathy scores for each emotion were also tested. These revealed no differences in terms of cognitive empathy, but differences were found in affective empathy for sadness, fear and affective empathy overall. In all cases, means for females were higher, suggesting better affective empathy in girls compared to boys. The % of participants who exceeded the threshold for clinically relevant anxiety symptoms according to the SCARED (≥ 25) was 28.4%.

**Table 1:** Descriptive statistics (*N* = 178)

	Whole Sample	Males	Females	Test Statistic (Males vs. Females)				
	(*N* = 178)	(*n* = 115)	(*n* = 63)	*t*	*p*			
	Mean (SD)	Mean (SD)	Mean (SD)			Range	Skewness	Kurtosis
Gender	64.6% Male							
Age	77.17 (11.56)	76.28 (11.85)	78.79 (10.93)	1.39	0.17	51–98	-0.26	-0.77
Income (% less than £20,000 pa)	25.30%							
Race								
White	94%							
Black	4%							
South Asian	2%							
Ethnicity								
British	94%							
African	3%							
Bangladeshi/Pakistani	2%							
Caribbean	1%							
**Anxiety Score**								
Total SCARED	14.12 (10.39)	13.10 (9.73)	15.94 (11.31)	1.67	0.1	0–47	1.06	1.19
**Empathy**								
**Cognitive Empathy**								
Happy	5.44 (2.19)	5.40 (2.09)	5.52 (2.36)	0.36	0.72	1–8	-0.48	-1.05
Sad	3.70 (2.30)	3.46 (2.23)	4.13 (2.36)	1.83	0.07	1–8	0.48	-0.98
Fear	3.38 (2.18)	3.29 (2.28)	3.54 (2.01)	0.71	0.48	0–8	0.77	-0.45
Total	12.56 (5.21)	12.20 (5.10)	13.18 (5.38)	1.17	0.24	3–24	0.02	-0.94
**Affective Empathy**								
Happy	4.27 (1.91)	4.08 (1.91)	4.62 (1.89)	1.78	0.08	0–6	-0.76	-0.55
Sad	1.77 (1.89)	1.46 (1.77)	2.33 (1.99)	2.93	< 0.01*	0–6	0.71	-0.64
Fear	1.40 (1.85)	1.02 (1.74)	2.08 (1.87)	3.71	< 0.001**	0–6	1.08	-0.01
Total	7.40 (4.12)	6.50 (4.03)	8.98 (3.81)	3.92	< 0.001**	0–17	0.3	-0.75
**CBCL-rated Psychopathology Symptoms (t-score)_**								
Externalising	58.08 (10.43)	58.38 (10.43)	57.91 (10.47)	0.29	0.78	32–83	-0.03	-0.25
Internalising	56.23 (9.98)	55.03 (10.20)	56.88 (9.85)	1.18	0.24	33–85	-0.25	0.13

*Note* * *p* < .01; ** *p* < .001

Our sample showed a mean of 56.23 for CBCL-rated internalising symptoms. Of these, 58.2% had a t-score of ≤ 59 indicating non-clinical symptoms, 23.1% had a t-score of between 60 and 64 (borderline / at-risk), and 18.7% had a t-score of 65 or above (clinically significant symptoms). Additionally, our sample showed a mean of 58.08 for CBCL-rated externalising symptoms. Of these, 55.6% had a t-score of ≤ 59 indicating non-clinical symptoms, 20.8% had a t-score of between 60 and 64 (borderline / at-risk), and 23.6% had a t-score of 65 or above (clinically significant symptoms).

Correlations between SCARED total and CBCL internalising and externalising scores were tested for (thresholds = 0.00 − 0.49 low; 0.50 − 0.69 moderate; ≥0.70 high; Hinkle et al., 2003). SCARED total scores were positively associated with internalising scores (*r*_s_ = 0.60, *p* < .001) but not with externalising scores (*r*_s_ = − 0.02, *p* = .76). Despite a moderate correlation between SCARED total scores and CBCL internalising scores, SCARED total scores were used as our measure of anxiety as we wanted to examine specific effects of anxiety, but not other factors clustered underneath the umbrella term ‘internalising disorders’, such as depression. Because the sample was referred to the NDAU for behavioural problems, all correlational analyses were carried out whilst controlling for the influence of externalising symptoms.

### Main Analyses

Table 2 reports associations between total anxiety scores and cognitive and affective empathy. Anxiety was significantly negatively related to cognitive empathy for all emotions, and to total cognitive empathy (*r* = −.30, *p* < .001), but was not associated with affective empathy.

**Table 2:** Associations between cognitive and affective empathy and SCARED scores, controlling for externalising symptoms

	Total SCARED score	
	*r*	*p*
Cognitive Empathy		
Sad (Fish)	-0.25*	0.002
Happy (Christmas)	-0.21*	0.01
Fear (Carwash)	-0.26*	0.002
Total	-0.30**	< 0.001
Affective Empathy		
Sad (Fish)	0.07	0.40
Happy (Christmas)	0.02	0.86
Fear (Carwash)	0.05	0.53
Total	0.06	0.46

*Note* * *p* ≤.01, ** *p* < .001

### Eye-Tracking Metrics – Anxiety and Social Attention

As anxiety was found to be associated with empathy scores, we ran tests for correlations between eye-tracking data and anxiety for each emotion (see Table 3). Given the number of tests performed, a Bonferroni adjusted alpha level (α = 0.01) was used to identify any significant associations. For the happy emotion condition, total anxiety scores were negatively associated with first fixation duration (*r* = −.61, *p* < .001), total fixation duration (*r* = −.24, *p* = .004) and fixation count (*r* = −.60, *p* < .001), but not associated with time to first fixation. For the sad emotion condition, total anxiety scores were negatively associated with first fixation duration (*r* = −.35, *p* < .001) and total fixation duration (*r* = −.49, *p* < .001), and positively associated with fixation count (*r* = .21, *p* = .01), but were not associated with time to first fixation. For the fear emotion condition, anxiety was positively associated with time to first fixation (*r* = .54, *p* < .001), and negatively associated with first fixation duration (*r* = −.48, *p* < .001) and total fixation duration (*r* = −.31, *p* < .001), but not associated with fixation count.

**Table 3:** Associations between anxiety and ET variables, controlling for externalising symptoms

	Total SCARED score		
	*r*	*p*	
Happy (Christmas)			
TTFF	0.10	0.25	
FFD	-0.61**	< 0.001	
TFD	-0.24*	0.004	
FC	-0.60**	< 0.001	
Sad (Fish)			
TTFF	0.11	0.18	
FFD	-0.35**	< 0.001	
TFD	-0.49**	< 0.001	
FC	0.21*	0.01	
Fear (Carwash)			
TTFF	0.54**	< 0.001	
FFD	-0.48**	< 0.001	
TFD	-0.31**	< 0.001	
FC	-0.05	0.58	

*Note* * *p* ≤.01, ** *p* < .001; TTFF = Time to First Fixation; FFD = First Fixation Duration; TFD = Total Fixation Duration; FC = Fixation Count

### Eye-Tracking Metrics – Empathy and Social Attention

Correlations between eye-tracking metrics and cognitive and affective empathy were then tested for (see Table 4). As above, given the high number of tests performed, a Bonferroni adjusted alpha level (α = 0.001) was used to identify any significant associations.

**Table 4:** Associations between cognitive and affective empathy for all emotions and ET variables, controlling for externalising symptoms

	Cognitive Empathy								Affective Empathy							
	Happy (Christmas)		Sad (Fish)		Fear (Carwash)		Total		Happy (Christmas)		Sad (Fish)		Fear (Carwash)		Total	
	*r*	*p*	*r*	*p*	*r*	*p*	*r*	*p*	*r*	*p*	*r*	*p*	*r*	*p*	*r*	*p*
Happy (Christmas)																
TTFF	-0.23	0.01	-0.11	0.17	-0.08	0.33	-0.18	0.03	0.17	0.03	-0.01	0.94	0.06	0.50	0.10	0.21
FFD	0.35**	<0.001	0.37**	<0.001	0.31**	<0.001	0.44**	<0.001	-0.12	0.15	-0.02	0.86	-0.15	0.07	-0.13	0.11
TFD	0.08	0.33	0.15	0.07	0.21	0.01	0.19	0.02	0.01	0.89	0.17	0.04	0.28**	<0.001	0.21	0.01
FC	0.27**	<0.001	0.24	0.00	0.29**	<0.001	0.34**	<0.001	-0.21	0.01	-0.09	0.27	-0.19	0.02	-0.23	0.01
Sad (Fish)																
TTFF	0.03	0.74	0.15	0.08	0.03	0.71	0.09	0.29	0.10	0.22	0.17	0.04	0.16	0.06	0.20	0.02
FFD	0.18	0.03	0.38**	<0.001	0.23	0.01	0.34**	<0.001	0.01	0.94	0.41**	<0.001	0.15	0.07	0.26*	0.00
TFD	0.23	0.00	0.37**	<0.001	0.32**	<0.001	0.40**	<0.001	0.10	0.24	0.19	0.02	0.08	0.35	0.17	0.04
FC	0.03	0.70	0.12	0.15	0.00	0.99	0.06	0.36	0.13	0.12	0.17	0.04	0.19	0.03	0.22	0.01
Fear (Carwash)																
TTFF	-0.30**	<0.001	-0.27**	<0.001	-0.26*	0.001	-0.36**	<0.001	0.22	0.01	0.06	0.47	0.15	0.06	0.20	0.01
FFD	0.25	0.00	0.32**	<0.001	0.29**	<0.001	0.37**	<0.001	0.19	0.02	0.09	0.27	-0.05	0.57	0.11	0.18
TFD	0.17	0.04	0.07	0.42	0.22	0.01	0.19	0.02	-0.14	0.09	-0.03	0.69	-0.04	0.67	-0.10	0.23
FC	0.11	0.16	0.10	0.22	0.11	0.18	0.14	0.09	-0.03	0.69	-0.04	0.67	-0.05	0.55	-0.06	0.50

*Note* * *p* = .001, ** *p* ≤.001; TTFF = Time to First Fixation, FFD = First Fixation Duration, TFD = Total Fixation Duration, FC = Fixation Count

### Cognitive Empathy

Total cognitive empathy scores were positively associated with first fixation duration variables across all three emotions. First fixation duration (*r* = .35, *p* < .001) and fixation count (*r* = .27, *p* < .001) were positively associated with cognitive empathy for happiness. Similarly, first fixation duration (*r* = .38, *p* < .001) and total fixation duration (*r* = .37, *p* < .001) were positively associated with cognitive empathy for sadness. Finally, first fixation duration (*r* = .29, *p* < .001) was positively associated, and time to first fixation (*r* = −.36, *p* < .001) negatively associated, with cognitive empathy for fear. With the exception of the fear emotion condition, all significant associations between cognitive empathy and eye-tracking metrics were positive, suggesting that greater social attention was associated with better cognitive empathy.

### Affective Empathy

Total affective empathy scores were positively associated with first fixation duration (*r* = .26, *p* = .001) when viewing the sad emotion only. Affective empathy for sadness was also associated with first fixation duration when viewing the sad emotion (*r =*.41, *p* < .001), and affective empathy for fear was positively associated with total fixation duration when viewing the happy emotion (*r* = .28, *p* < .001).

### Effects of Age, Gender, Empathy, and Gaze Patterns on Anxiety Symptoms

Age, gender, externalising scores, SCARED total scores, and cognitive and affective empathy total scores, were entered into three hierarchical multiple regression analyses, one for each emotion (happiness, sadness, and fear) to better understand the impact of empathy and social attention in predicting anxiety severity in children (Table 5).

**Table 5:** Hierarchical multiple regression analyses examining the contributing effects of first fixation duration and cognitive and affective empathy on total anxiety scores, controlling for age, gender, and externalising symptoms

Step	Predictor	Standardised β	Sig.	R2	ΔR2	Sig. Change
**Fixation – Happy**						
1	Gender	0.11	0.18			
	Age	-0.05	0.53			
	Externalising Disorders	0.05	0.59	0.02	0.02	0.50
2	Gender	0.04	0.61			
	Age	-0.03	0.66			
	Externalising Disorders	0.11	0.10			
	First Fixation Duration	-0.60**	< 0.001	0.37	0.35**	< 0.001
3	Gender	0.04	0.59			
	Age	-0.02	0.75			
	Externalising Disorders	0.12	0.10			
	First Fixation Duration	-0.59**	< 0.001			
	Cognitive Empathy Total	-0.02	0.80	0.37	< 0.001	0.80
4	Gender	0.04	0.59			
	Age	-0.02	0.75			
	Externalising Disorders	0.12	0.10			
	First Fixation Duration	-0.59**	< 0.001			
	Cognitive Empathy Total	-0.02	0.84			
	Affective Empathy Total	− 0.001	0.99	0.37	< 0.001	0.99
**Fixation – Sad**						
1	Gender	0.12	0.17			
	Age	-0.05	0.56			
	Externalising Disorders	0.06	0.45	0.02	0.02	0.44
2	Gender	0.13	0.11			
	Age	0.001	0.99			
	Externalising Disorders	0.08	0.33			
	First Fixation Duration	-0.36**	< 0.001	0.15	0.13**	< 0.001
3	Gender	0.14	0.08			
	Age	0.07	0.40			
	Externalising Disorders	0.11	0.17			
	First Fixation Duration	-0.29**	< 0.001			
	Cognitive Empathy Total	-0.23*	0.01	0.19	0.04*	0.01
4	Gender	0.08	0.31			
	Age	0.08	0.32			
	First Fixation Duration	-0.33**	< 0.001			
	Externalising Disorders	0.10	0.19			
	Cognitive Empathy Total	-0.33**	< 0.001			
	Affective Empathy Total	0.26*	0.003	0.24	0.05*	0.003
**Fixation – Fear**						
1	Gender	0.12	0.16			
	Age	-0.07	0.42			
	Externalising Disorders	0.03	0.69	0.02	0.02	0.46
2	Gender	0.07	0.35			
	Age	0.06	0.43			
	Externalising Disorders	0.09	0.23			
	First Fixation Duration	-0.47**	< 0.001	0.23	0.21**	< 0.001
3	Gender	0.08	0.29			
	Age	-0.01	0.88			
	Externalising Disorders	0.10	0.18			
	First Fixation Duration	-0.42**	< 0.001			
	Cognitive Empathy Total	-0.14	0.12	0.25	0.01	0.12
4	Gender	0.04	0.64			
	Age	-0.01	0.94			
	First Fixation Duration	-0.42**	< 0.001			
	Externalising Disorders	0.10	0.19			
	Cognitive Empathy Total	-0.22	0.02			
	Affective Empathy Total	0.21	0.02	0.28	0.03	0.01

*Note* * *p* < .0125, ** *p* < .001

First fixation duration was selected as an index of gaze pattern, as this metric was found to be consistently negatively associated with total anxiety scores across all three emotions. Tolerance and VIF values for all regression analyses suggested no evidence of multicollinearity (tolerance ranged from 0.49 − 0.99, and VIF ranged from 1.01 to 2.06), indicating that it was acceptable to run the proposed analyses with these data. Age, gender, and externalising symptoms were entered at Step 1 as variables that were controlled for, followed by First Fixation Duration at Step 2, cognitive empathy at Step 3, and finally affective empathy at Step 4. A Bonferroni adjusted alpha level (α = 0.0125) was used to identify any significant associations between variables. The first models examining effects for happiness, sadness, and fear were not significant, suggesting that gender, age, and externalising symptoms were not predictive of anxiety symptoms across all emotion conditions.

The second models, that included the first fixation duration metric, all showed significant improvement from the first models. The second model for the happy emotion condition was significant (*F*(4, 139) = 19.94, *p* < .001), with the addition of the first fixation duration variable accounting for an additional 35% of the variance in anxiety (ΔR2 = 0.35, *F* change(1, 139) = 76.31, *p* < .001). The second model for sadness was significant (*F*(4, 138) = 5.83, *p* < .001), with the addition of the first fixation duration variable accounting for an additional 13% of the variance in anxiety (ΔR2 = 0.13, *F* change(1, 138), *p* < .001). The second model for the fear emotion condition was also significant (*F*(4, 137) = 10.31, *p* < .001), with the addition of the first fixation duration variable accounting for an additional 21% of the variance in anxiety (ΔR2 = 0.21, *F* change(1, 137) = 37.90, *p* < .001). These improvements suggest that social attention (in the form of first fixation duration) negatively predicted anxiety symptoms across all emotion conditions, with the largest effect size seen for the happiness emotion (β = − 0.60). The addition of cognitive and affective empathy did not significantly improve the model for happiness. When added to the model for sadness, both cognitive (ΔR2 = 0.04, *F* change(1, 137) = 6.79, *p* = .01) and affective (ΔR2 = 0.05, *F* change(1, 136) = 9.31, *p* = .003) empathy significantly improved the model. For the fear condition, the addition of cognitive empathy did not significantly improve the model, though the addition of affective empathy did (ΔR2 = 0.03, *F* change(1, 135) = 6.05, *p* = .01).

## Discussion

The current study used an RDoC approach to examine associations between social attention, empathy, and anxiety in a sample of young children with emerging mental health problems. Under the RDoC framework, this study is an examination of the potential threat (anxiety) construct, which resides under the negative valence domain(Insel et al., 2010). We found that, when controlling for externalizing symptoms, children more sensitive to potential threats (anxiety) displayed less cognitive empathy for happiness, sadness, and fear, suggesting that they show impairments in recognizing and understanding the emotions of others.

Additionally, eye-tracking metrics pointed to a persistent pattern of shorter first fixation durations on the eyes across all emotion conditions in those higher in anxiety, when controlling for externalizing symptoms. Conversely, those who were better in cognitive empathy displayed longer first fixation durations across all emotion conditions. These findings point to a potential link between cognitive empathy and anxiety in children, with those attending longer on the eyes of those displaying happiness, sadness, or fear, being better in cognitive empathy and lower in anxiety. No such associations were found between anxiety and affective empathy.

Our first hypothesis, that children with higher levels of anxiety would show greater impairments in empathy, was supported with regards to cognitive, but not affective, empathy. The lack of associations found between affective empathy and anxiety contrasts with the findings of Gambin and Sharp (2018) and Pittelkow et al. (2021) and could be due to differences in neural and cognitive mechanisms related to each empathy subtype. Broadly, cognitive empathy is a top-down process that requires an individual to make inferences about the thoughts and beliefs of others through a change of perspective, similar to Theory of Mind (Preckel et al., 2018), whereas affective empathy is a bottom-up process in which an individual models the emotion they have inferred (de Waal, 2008).

Therefore, although affective empathy also requires changes in perspective from one’s current emotional state to one that resembles another person’s, it also includes an emotion processing component. Accordingly, evidence from neuroscientific studies suggests that cognitive and affective perspective taking recruit different areas of the brain, with cognitive perspective taking using frontal areas and affective perspective taking using frontal *and* limbic areas (Healey & Grossman, 2018). As such, the findings of the present study could be showing impairments in perspective taking (i.e., cognitive empathy), but not in emotion processing (i.e., affective empathy).

Cognitive empathy for all emotions was found to be negatively related to anxiety scores, suggesting that those higher anxiety were lower in cognitive empathy. Cognitive empathy is believed to rely on various cognitive control processes (Carlson et al., 2004). Correspondingly, Yan et al. (2020) found that cognitive empathy was related to inhibitory control, working memory, and cognitive flexibility, whereas affective empathy was related to inhibitory control only. These prior empirical studies suggest that an examination of executive functioning and emotional processing ability, in a similar sample, could further define these relationships and explain the present findings. Such analyses could be investigated under the ‘cognitive systems’ domain of the RDoC framework, contributing further to the transdiagnostic literature.

Our second hypothesis regarding eye gaze patterns, was that children higher in anxiety and lower in empathy would spend less time attending to the eyes when viewing negative stimuli was supported. In the case of anxiety, we found anxiety symptoms to be negatively correlated with attention to the eyes, indexed by first fixation duration and total fixation duration across the emotion conditions. Additionally, we found that fixation count was negatively associated with anxiety in the happy condition. With regards to gaze patterns and empathy, we found that those higher in cognitive empathy showed longer first fixation duration and greater total fixation duration during the sad condition, and longer first fixation duration and shorter time to first fixation for the fear condition. This suggests that those higher in cognitive empathy could attend to the eyes of those experiencing negative emotions better than those lower in cognitive empathy. With regards to affective empathy, we found positive associations between first fixation duration and affective empathy for sadness and total affective empathy only.

A hierarchical regression analyses showed that when controlling for age, gender, and externalizing symptoms, eye gaze patterns and cognitive empathy scores negatively predicted, but affective empathy scores positively predicted, anxiety scores during fear processing. For the sad condition, first fixation duration and cognitive empathy negatively predicted anxiety at equivalent strengths, and affective empathy positively predicted anxiety but to a lesser degree. For the happy film clip, first fixation duration alone predicted anxiety; however, the final overall model for this clip explained the most variance (37%).

This pattern of findings suggests that eye gaze patterns were consistently negatively associated with anxiety, regardless of emotional condition. However, although not all findings reached significance across the negative emotion conditions, both first fixation duration and cognitive empathy *negatively* predicted anxiety, whilst affective empathy *positively* predicted it. This suggests that those higher in cognitive empathy, and who spent less time fixating on the eyes of the protagonist in the clip initially, were lower in anxiety, whereas those higher in affective empathy were also higher in anxiety. As anxiety symptoms and affective empathy scores showed equivalent directional effects, they support the proposition by Gambin and Sharp (2018), that displays of anxiety can enhance anxious states in the observer. These findings contrast with the results from a previous study from our lab (Howe-Davies et al., 2022), which found negative associations between anxiety and affective empathy. The discrepancy between these findings may be attributed to the use of different measures of anxiety between the studies. Howe-Davies et al. study used a composite measure of anxiety dimensions derived from a clinical interview (i.e., the Development and Well-Being Assessment (DAWBA); Goodman et al., 2000), whereas the present study used a screening questionnaire (i.e., the SCARED scale).

### Strengths and Limitations

The current study examines the role of anxiety in social-emotional functioning through a dimensional lens, in line with observations that psychosocial problems in children are heterogenic and vary in severity. The study benefitted from the use of dynamic stimuli, meaning that social attention was assessed using stimuli that more closely resembles real life events, and which are therefore more likely to provoke emotional responses than static stimuli (e.g., grayscale images). Additionally, we controlled for externalizing symptoms in our analyses, which reduces the possibility that our observed effects could be attributed to other psychopathologies in the sample. Furthermore, 58.2% of the sample had a CBCL-rated internalizing t-score of ≤ 59 (indicating non-clinical symptoms), with the rest of the sample either borderline / at-risk (23.1%) or showing clinical symptoms (18.7%). This illustrates variability within the sample, strengthening the findings.

Nevertheless, the present study also has several limitations. Firstly, the cross-sectional study design means we cannot make inferences of causality. That is, it is not clear from our findings if sensitivity to threat negatively impacts upon the development of empathy (cognitive or affective) or if poor empathic responses engender symptoms of anxiety. Alternatively, it may be that anxiety impairs an individual’s social attention, or that poor social attention leads to a failure to understand emotions in others, which creates anxiety. Secondly, anxiety measures in our study were reported by the parent and these could have been either exaggerated or understated. Thirdly, related to this, our measures of cognitive and affective empathy were reported by the children. Assessments of empathy through self-reporting can be confounded by bias, in that the child may have felt compelled to state that they felt something rather than nothing when witnessing the protagonists in the videos displaying intense emotions (Deuter et al., 2018). Physiological measures (i.e., motor empathy; van der Graaf et al., 2016) could more objectively capture the extent to which children are emotionally affected by the stimuli. Fourthly, the sample was predominantly white (945). This lack of diversity within the sample could limit the extent to which the findings can be generalized.

### Implications and Future Directions

The observed negative relationship between cognitive empathy and threat sensitivity suggests that children high in anxiety could be educated about ways to correctly identify the emotions they observe in daily life, which in turn could help to reduce threat sensitivity and subsequently, ameliorate their anxiety symptoms. This could be tested by encouraging children high in anxiety to focus on relevant facial stimuli (i.e., the eyes) to improve their social attention. If improved social attention is found to improve cognitive empathy, our study suggests that this could in turn mitigate symptoms of anxiety. Future research could therefore investigate whether teaching children high in anxiety to increase social attention to the eyes (both positive and negative) improves emotion recognition ability, and in turn, increases cognitive empathy and decreases threat sensitivity. This proposal is supported by previous research using the Cardiff Emotion Recognition Training (CERT) paradigm which has shown that improving emotion recognition in children by teaching them about specific areas of the face that can reveal differences between emotion, subsequently improves emotional functioning and longer-term behavioral problems (Hunnikin et al., 2022; Wells et al., 2021). Furthermore, research into the emotional processing component of affective empathy, and cognitive and emotional set-shifting (‘hot’ and ‘cool’ executive functioning), in children with emerging mental health problems could shed light on associations between anxiety and affective empathy and whether these associations are related to perspective taking or emotion processing. Finally, the RDoC initiative provides a framework for studying psychopathology using biological and behavioral components measured dimensionally and across domains of functioning (Insel et al., 2010). Whilst the present study adheres to this approach, it does so using the ‘physiology’ (eye-tracking) and ‘self-report’ (empathy quotient) units of analyses to investigate the construct of ‘Potential threat’ (anxiety) under the ‘Negative Valence Systems’ domain. Future studies should use measures corresponding to other units of analyses within the RDoC framework, such as cortisol (‘molecular’ unit of analysis) or fronto-amygdala circuitry function (‘circuits’ unit of analysis), in order to align with the RDoC principles of integrative science and provide a more comprehensive understanding of the relationship between these constructs.

## Conclusions

Overall, our results suggest that children higher in anxiety are more likely to show cognitive empathy and social attention impairments. Failing to successfully recognize emotional states in others means that some children may find themselves unable to respond appropriately to a given situation, which could increase symptoms of anxiety. We also found that, when controlling for age, gender, and externalizing symptoms, eye-gaze patterns and cognitive and affective empathy work in divergent ways when observing fear and sadness, with eye-gaze patterns and cognitive empathy negatively predicting, and affective empathy positively predicting, anxiety. Due to the sample and measures used, this study uniquely contributes to other findings in the literature that highlight possible relationships between social attention, cognitive and affective empathy, and anxiety symptoms.

## Electronic Supplementary Material

Below is the link to the electronic supplementary material.

Supplementary Material 1

## Data Availability

The conditions of our ethics approval do not permit public archiving of anonymized study data. Access to the data can be requested by contacting the lead investigator (SvG) or the local ethics committee at the School of Psychology, Cardiff University. Access will be granted to named individuals in accordance with ethical procedures governing the reuse of sensitive data. Specifically, requestors must complete a formal data sharing agreement with the lead investigator.
