# Supplementary figures and images for: Impaired Social Attention and Cognitive Empathy in a Paediatric Sample of Children with Symptoms of Anxiety
Source: Res Child Adolesc Psychopathol. 2024 Sep 18;52(12):1945–60. doi: 10.1007/s10802-024-01240-7 (PMC11624222; doi:10.1007/s10802-024-01240-7)

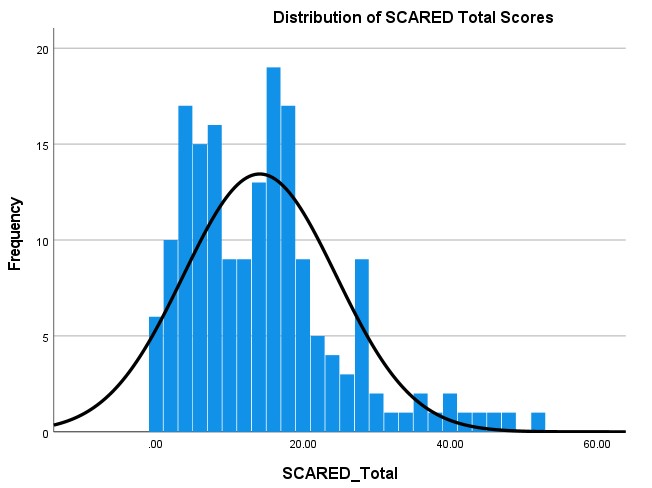


*Supplementary Figure 1 – Distribution of SCARED Total Scores*

Supplement: Supplementary file 1 — Supplementary Material 1 [file 10802_2024_1240_MOESM1_ESM.docx]
